# Clinicopathological significance of KAI1 expression and epithelial-mesenchymal transition in non-small cell lung cancer

**DOI:** 10.1186/s12957-015-0657-8

**Published:** 2015-08-01

**Authors:** Lei Zhou, Lan Yu, Shiwu Wu, Zhenzhong Feng, Wenqing Song, Xiaomeng Gong

**Affiliations:** Department of Pathology, the First Hospital Affiliated of Bengbu Medical College, Bengbu Medical College, No. 287 Changhuai Ave, Longzihu, Bengbu, Anhui Province 233003 China

**Keywords:** KAI1, Epithelial-mesenchymal transition, Angiogenesis, Lymphangiogenesis, NSCLC

## Abstract

**Background:**

KAI1 and epithelial-mesenchymal transition (EMT) is related to both angiogenesis and lymphangiogenesis and is an important target in new cancer treatment strategies. We aimed to investigate the KAI1 and marker of EMT expression and correlation with lymph node metastasis (LNM) and explore their prognostic impact in non-small cell lung cancer (NSCLC).

**Methods:**

Tumor tissue specimens from 312 resected patients with stage I–IIIA NSCLC were obtained. Immunohistochemistry was used to assess the expression of the molecular markers KAI1, E-cadherin (E-cad), vimentin, CD34, and D2-40.

**Results:**

There were 153 N0 and 159 N+ patients. Tumor cell expression of KAI1and the marker of EMT, lymphatic vessel density (LVD), and microvessel density (MVD) were related to LNM. In multivariate analyses, the ages of patients, high tumor cell KAI1 expression, EMT, and the scores of MVD were independent factor of prognosis.

**Conclusions:**

Tumor cell KAI1 expression, EMT, LVD, and MVD correlate with LNM. Thus, the detection of KAI1, expression of markers of EMT, and the scores of MVD may be used as a potential indicator of NSCLC prognosis.

## Background

Lung cancer is the most frequent malignancy and the first leading cause of cancer-related death worldwide [1]. Complete surgical resection is the only potential curative treatment for localized lung cancer. About 65 % non-small cell lung cancer (NSCLC) patients who are suitable for surgical procedures will relapse within 2 years and subsequently die of metastatic spread [2, 3]. A common feature of tetraspanins is regulating cell motility. The mechanism that tetraspanins modulate cell motility is still unclear. KAI1 gene was originally identified as a suppressor of metastasis of tumor in prostate cancer and located on human chromosome 11p11.2 [4]. It is a member of the tetraspanins superfamily of glycoproteins. KAI1 suppresses metastasis by multiple mechanisms regulating inhibition of cell motility, adhesion, fusion, and proliferation [5]. It was supposed that KAI1 exerted its function by modulating membrane structure by interactions with cell surface molecules, such as cell adhesion molecules and other tetraspanins. Many studies have shown that decreased KAI1 expression could be a useful marker for metastatic, invasive, and prognostic factor in many human tumors, such as lung [6], breast [7, 8], gastric [9, 10], liver [11], colorectal [12], bladder[13], esophageal [14], and prostatic cancer [15].

Epithelial-mesenchymal transition (EMT) is a reversible embryonic process and aberrantly activated in tumor progression and metastasis. Many studies have shown that EMT was a critical process in tumor invasion and metastasis in NSCLC [16, 17]. It is commonly characterized by downregulation of E-cadherin (E-cad) which is a critical cell to cell adhesion molecule [18] and upregulation of vimentin which plays an important role in cell migration [19]. KAI1 may stabilize or strengthen E-cad-dependent cell-to-cell adhesion and motility by regulating β-catenin-mediated signal transduction on tumor cells, thus preventing tumor cells from seceding from the primary tumor [20]. These effects suggest that KAI1-induced E-cad signaling provides a survival benefit for metastatic tumors; however, whether KAI1 can inhibit EMT in NSCLC cells and the mechanism involved remains unclear.

## Methods

### Patients and clinical samples

Primary tumor tissues diagnosed NSCLC with pathologic stage I–IIIA from patients at the First Hospital Affiliated of Bengbu Medical College from 2003 to 2007 were used in this retrospective study. In total, 365 patients were registered from the hospital database. Of these, 53 patients were excluded from the study due to the following: (i) radiotherapy or chemotherapy before surgery (*n* = 15), (ii) other malignancy within 5 years before NSCLC diagnosis (*n* = 17), and (iii) inadequate paraffin-embedded fixed tissue blocks (*n* = 20). Thus, 312 patients with complete medical records and adequate paraffin-embedded tissue blocks were eligible. Due to financial or other reasons, 265 (84.9 %) patients were to postoperative therapy (postoperative routine chemotherapy or radiotherapy).

This report includes follow-up data as of 31 October 2012. The median follow-up was 42 (range 8–105) months. All specimens were obtained from the archives of the Department of Pathology at our hospital. The tumors were graded according to the World Health Organization and staged according to the International Union Against Cancer’s tumor-node-metastasis classification. This study was approved by the ethical committee of Bengbu Medical College before it started.

### Immunohistochemistry

All applied antibodies were subjected for immunohistochemistry (IHC) analysis on paraffin-embedded tissue. The antibodies used in this study were as follows: KAI1 (mouse monoclonal; Santa Cruz), E-cad (mouse monoclonal; LabVision), vimentin (mouse monoclonal; LabVision), CD34 (mouse monoclonal; LabVision), and D2-40 (mouse monoclonal; LabVision). The IHC procedures for these markers were previously described [21]. For each antibody, including negative controls, IHC was done in a single experiment.

### Scoring of IHC

By light microscopy, representative tissue sections were scored semiquantitatively for cytoplasmic and membrane staining. All samples were anonymized and independently observed by two pathologists. If there is a disagreement, the observers would reexamine and reach a consensus. In scoring expression of antibodies, both the intensity and extent of immunopositivity were considered. The dominant staining intensity in tumors was scored as follows: 0 = negative, 1 = weak, 2 = moderate, and 3 = strong. The extent of positive staining tumor cells was scored as follows: <10 % is 1, 11–50 % is 2, 51–75 % is 3, and >75 % is 4. The final score was determined by multiplying the intensity and the extent positivity scores, which yielded a range from 0 to 12. Mean score from each individual was calculated in tumor cells. The positive expression for markers was scored as follows: >2, KAI1; >3, E-cadherin; and >1, vimentin.

The positive expression of KAI1 and E-cad was found mainly on the membrane and cytoplasm of NSCLC cells and normal lung tissues. The positive expression of vimentin was found mainly on the cytoplasm of NSCLC cells and normal lung tissues. They were presented as a brown granular material.

### Microvessel and lymphatic vessel density

We assessed lymphatic vessel density (LVD) by D2-40 and microvessel density (MVD) by CD34 immunohistochemical staining. Not only stained endothelial cell but also endothelial cell cluster separated from other stromal elements was considered as a countable microvessel or lymphatic vessel.

### Statistical methods

All statistical analyses were done using the statistical software SPSS (SPSS inc., Chicago, IL), version 17. The Fisher’s exact test and Pearson chi-square test for trends in proportions, Spearman’s correlate analysis, and Kaplan-Meier’s method with log-rank test or Cox regression method for univariate or multivariate OS or disease-specific survival (DSS) analysis were used to assess the associations among the positive staining of KAI1, E-cad, vimentin, LVD, or MVD and clinicopathological indices. DSS was defined from the date of surgery to the time of NSCLC death. A value of *P* < 0.05 was considered statistically significant.

## Results

### The relationship between expression of KAI1, E-cad, and vimentin and clinicopathological parameters

**Fig. 1:**
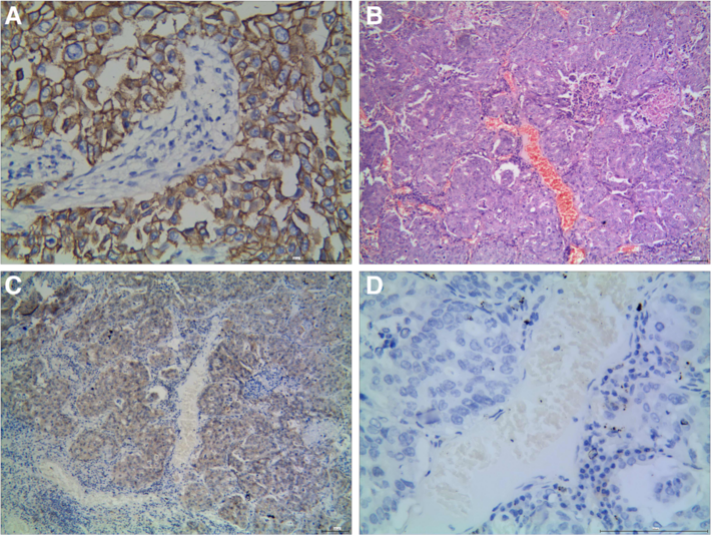
Representative results of KAI1 and vimentin and E-cadherin protein in NSCLC. **a** Moderately differentiated NSCLC, H&E staining. **b** KAI1 protein predominantly localized in the membrane and cytoplasm in well-differentiated squamous carcinoma. **c** NSCLC cells did not express a detectable level of E-cadherin protein. **d** Vimentin protein localized in the cytoplasm and membrane in NSCLC cells (**a**, **c**, and **d** are serial sections)

In NSCLC (Fig. 1b), the positive expression of KAI1 was 34.0 % (106/312) (Fig. 1a), the positive expression of E-cad was 39.7 % (124/312) (Fig. 1c), and the positive expression of vimentin was 42.3 %(132/312) (Fig. 1d). There was a significant difference between the expression of KAI1, E-cad, and vimentin and grades of tumors (*P* < 0.001) and TNM stage (*P* < 0.001) (Table 1).

**Table 1:** Correlation between KAI1 and vimentin and E-cad expression to clinicopathological characteristics in 312 NSCLC patients

Variable	KAI1		*P* value	E-cadherin		*P* value	Vimentin		*P* value
	(−)	(+)		(−)	(+)		(−)	(+)	
Ages			0.622			0.894			0.336
<60	95	52		88	59		89	58	
≥60	111	54		100	65		91	74	
Gender			0.038			0.423			0.597
Male	147	87		138	96		137	97	
Female	59	19		50	28		43	35	
Diameter			0.081			0.070			0.368
<3.0 cm	32	9		30	11		21	20	
≥3.0 cm	174	97		158	113		159	112	
Histology			0.283			0.077			0.323
SCC	155	79		144	90		137	97	
Adenocarcinoma	43	26		36	33		40	29	
LCC	8	1		8	1		3	6	
Smoking			0.957			0.791			0.185
Yes	151	78		139	90		127	102	
No	55	28		49	34		53	30	
Grades of tumor			<0.001			<0.001			0.002
Well	10	31		13	28		32	9	
Moderately	135	68		116	87		118	85	
Poorly	61	7		59	9		30	38	
TNM stages			<0.001			<0.001			<0.001
I	10	57		7	60		65	2	
II	85	35		77	43		89	31	
III	111	14		104	21		26	99	
VI			<0.001			<0.001			<0.001
Yes	42	1		40	3		5	38	
No	164	105		148	121		175	94	
Marginal free			0.119			0.085			<0.001
Yes	171	95		155	111		167	99	
No	35	11		33	13		13	33	

*NSCLC* non-small cell lung cancer, *SCC* squamous cell carcinoma, *LCC* large cell carcinoma, *VI* vascular invasion

### Expression of KAI1 and LNM and DSS (Table 2)

**Table 2:** KAI1 and EMT and vascular endothelial and lymphatic endothelial marker as predictors for lymph node metastasis in 312 NSCLC patients (*X* ^2^)

Marker expression	Patients (*n*)	N0 (*n*)	N1 (*n*)	N2 (*n*)	*P*(*X* ^2^)N0 Verse N1 Verse N2	*P*(*X* ^2^)N0 Verse N+	*P*(*X* ^2^) N0 + N1 Verse N2
KAI1					<0.001	<0.001	<0.001
Negative	206	84	80	42			
Positive	106	69	34	3			
Co-expression					<0.001	<0.001	<0.001
Low V/high E	114	75	36	3			
Low V/low E	66	29	33	4			
High V/high E	10	5	2	3			
High V/low E	122	44	43	35			
MVD score					<0.001	<0.001	<0.001
Mean	21.6 ± 12.0	18.0 ± 11.5	23.4 ± 11.0	29.2 ± 11.2			
LVD score					<0.001	0.005	<0.001
Mean	4.8 ± 2.8	4.4 ± 2.7	4.9 ± 2.7	6.2 ± 2.5			

The negative expression of KAI1 was seen significantly (*P* < 0.001) more often in N+ (50.5 %) than N0 when compared with positive expression of KAI1 (45.1 %).

The negative expression of KAI1 was seen significantly (*P* < 0.001) more frequent in N2 (14.4 %) than in N0 + N1 when compared with positive expression of KAI1 (38.6 %).

**Fig. 2:**
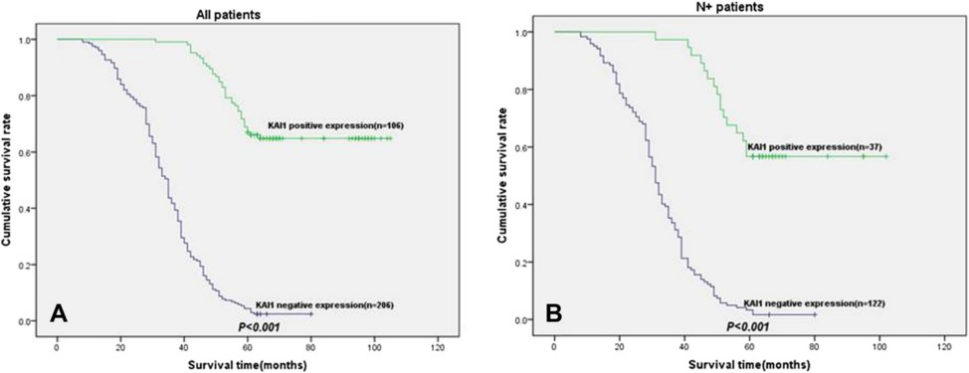
Disease-specific survival curves according to NSCLC cells expression of KAI1. **a** In the total cohort of patients (*n* = 312), the median survival in the negative expression of KAI1 group was 34.6 months, compared with 67.3 months in the positive expression of KAI1 group. **b** In all patients with N+ status (*n* = 159), the median survival in the negative expression of KAI1 group was 32.0 months, compared with 79.2 months in the positive expression of KAI1 group

In univariate analyses, the negative expression of KAI1 was clearly associated with poor survival (*P* < 0.001) as shown in Fig. 2a.

In the N+ (N1 + N2) patient cohort (159 patients), the negative expression of KAI1 group (*n* = 122) was related with a significantly worse prognosis (*P* < 0.001) when compared with the positive expression of KAI1 group (Fig. 2b).

### Co-expression of E-cad/vimentin and LNM (Table 2)

Low E-cad/high vimentin expression was seen significantly (*P* < 0.001) more often in N+ than N0 when compared with high E-cad/high vimentin (3.3 %), high E-cad/low vimentin (49.0 %), or low E-cad/low vimentin (19.0 %).

Low E-cad/high vimentin expression was seen significantly (*P* < 0.001) more frequent in N2 than in N0 + N1 when compared with high E-cad/high vimentin (2.6 %), high E-cad/low vimentin (41.6 %), or low E-cad/low vimentin (23.2 %).

### Co-expression of E-cad/vimentin and DSS

**Fig. 3:**
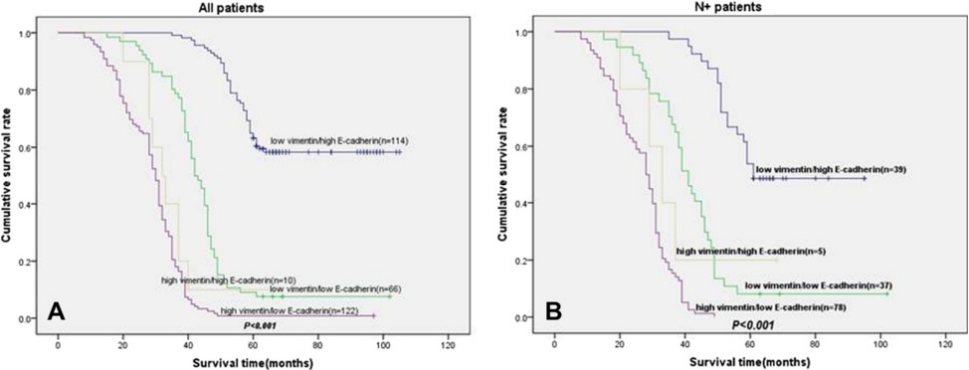
Disease-specific survival curves according to NSCLC cells co-expression of vimentin and E-cad. **a** In the total cohort of patients (*n* = 312), the median survival in the low E-cad/high vimentin group was 28.8 months, compared with 66.0 months in the high E-cad/low vimentin group and 43.3 months in the low E-cad/low vimentin group. **b** In all patients with N+ status (*n* = 159), the median survival in the low E-cad/high vimentin group was 48.4 months, compared with 69.1 months in the high E-cad/low vimentin group and 27.4 months in the low E-cad/low vimentin

In univariate analyses, the co-expression of low E-cad/high vimentin was clearly related with poor survival (*P* < 0.001) as shown in Fig. 3a.

In the N+ (N1 + N2) patient cohort (159 patients), the low E-cad/high vimentin expression group (*n* = 78) had a median survival of 26.7 months and was related with a significantly worse prognosis (*P* < 0.001) when compared with low E-cad/low vimentin and high E-cad/low vimentin expression group (Fig. 3b).

### LVD and MVD and LNM and DSS (Table 2)

High scores of LVD (5.2 ± 2.7) and MVD (25.0 ± 11.4) were seen significantly (*P* < 0.01) more often in N+ than N0 when compared with low scores of LVD (4.4 ± 2.7) and MVD (18.0 ± 11.5).

High scores of LVD (6.2 ± 2.5) and MVD (29.2 ± 11.2) were seen significantly (*P* < 0.001) more frequent in N2 than N0 + N1 when compared with low scores of LVD (4.6 ± 2.7) and MVD (20.3 ± 11.6).

**Fig. 4:**
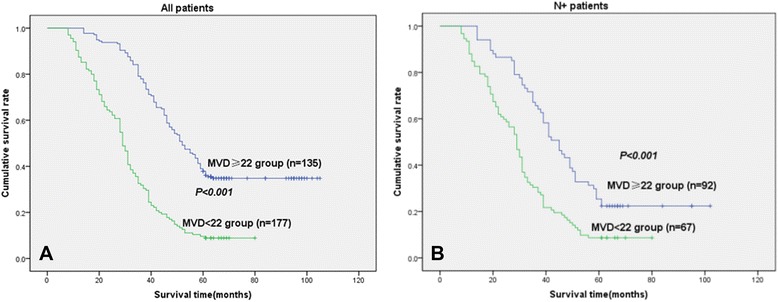
Disease-specific survival curves according to NSCLC of MVD. **a** In the total cohort of patients (*n* = 312), the median survival in the MVD <22 group was 57.1 months, compared with 31.7 months in the MVD ≥ 22 group. **b** In all patients with N+ status (*n* = 159), the median survival in the MVD <22 group was 52.4 months, compared with 31.8 months in the MVD ≥22 group (MVD ≥22, because the mean score of MVD was 21.6 ± 12.0)

**Fig. 5:**
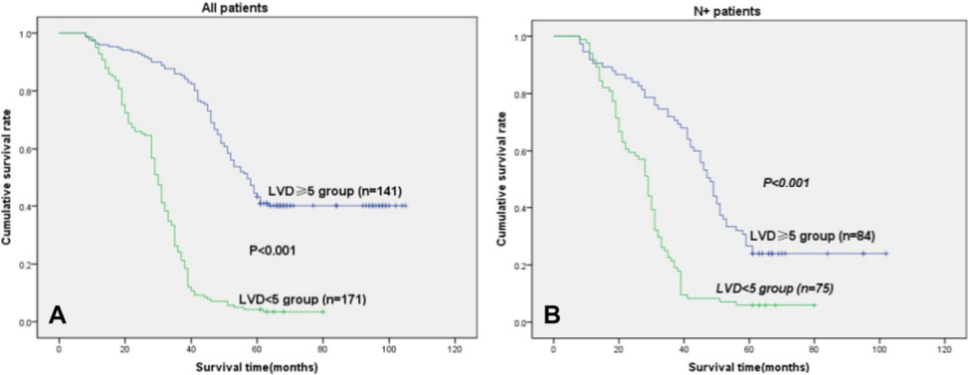
Disease-specific survival curves according to NSCLC of LVD. **a** In the total cohort of patients (*n* = 312), the median survival in the LVD <5 group was 54.5 months, compared with 34.0 months in the LVD ≥5 group. **b** In all patients with N+ status (*n* = 159), the median survival in the LVD <5 group was 53.7 months, compared with 29.4 months in the LVD ≥5 group (LVD ≥5, because the mean score of LVD was 4.8 ± 2.8)

In univariate analyses, the MVD ≥22 group or LVD ≥5 group was clearly associated with poor survival (*P* < 0.001) as shown in Fig. 4a or in Fig. 5a.

In the N+ (N1 + N2) patient cohort (159 patients), the MVD ≥22 group (*n* = 92) or LVD ≥5 group (*n* = 84) was related with a significantly worse prognosis (*P* < 0.001) when compared with the MVD <22 group (*n* = 67) or LVD <5 group (*n* = 75) (Fig. 4b or Fig. 5b).

In the multivariate Cox regression analysis, including all significantly clinicopathological parameters (Table 1) and the co-expression E-cad/vimentin and the scores of LVD and MVD, the co-expression of E-cad/vimentin, the expression of KAI1, the score of MVD, pTNM stages, age of patients, marginal, and therapy emerged as independent prognostic factors for DSS (Table 3).

**Table 3:** Multivariate survival analysis of 312 patients with NSCLC

Covariate	B	SE	Sig	Exp(B)	95 % CI
Age	−0.239	0.118	0.044	0.788	0.625–0.993
TNM stages	0.311	0.21	0.010	1.365	1.078–1.730
KAI1	−1.495	0.199	<0.001	0.224	0.152–0.332
MVD score	0.326	0.162	0.044	1.385	1.009–1.901
Marginal	1.091	0.176	<0.001	2.978	2.110–4.203
Co-expression	0.633	0.074	<0.001	1.882	1.628–2.176
Therapy	−0.551	0.170	0.001	0.576	0.413–0.804

## Discussion

In this study, we analyze 312 samples of surgically resected NSCLC Chinese patients using immunohistochemical method. We found that tumor cells expressed KAI1, E-cadherin, and vimentin protein to be independently associated with nodal metastasis and the co-expression of E-cadherin and vimentin and expression of KAI1 in tumor cells to be significantly associated with NSCLC patient prognosis.

To identify the relationship between KAI1 and EMT in NSCLC, five frequent indicator markers were detected in NSCLC tissues using IHC method. Many studies [7, 20, 22–24] have indicated that KAI1 could regulate E-cad-dependent cellular adhesion in many tumors. In this study, we found that KAI1 protein expression was decreased in NSCLC and was correlated with grade of tumors and a poor prognosis, and so was E-cad. There was a positive relationship between the expression of KAI1 and E-cad. The low expression of KAI1 and E-cad lost their function which inhibited tumor cells invasion. In vivo data suggested that KAI1 be involved in EMT of NSCLC. We also found that there was a negative correlation between KAI1 and vimentin. These indicated that there was a potential role of KAI1 in EMT of NSCLC. However, to our knowledge, there was no study available on the association between the expression of KAI1 and EMT. The mechanism might have been the hypothesis as follows: Cell adhesion and motility, the functions of E-cad, were commonly regulated by β-catenin which forms E-cad-β-catenin complexes. KAI1 could inhibit β-catenin tyrosine phosphorylation and stabilize E-cad-β-catenin complexes and thus prevent tumor cells dissemination from primary tumors [20]. The protein tyrosine kinase Src had been reported to be potent induces of EMT [25]. KAI1 expression could inhibit activation of Src kinase in the process of tumor malignancy and angiogenesis [26–28].

EMT has been found to be critical in tumor dissemination, local invasion, and metastasis, endowing cells with cancer stem cell properties [29–31]. Its common characteristic is downregulation of E-cad expression and upregulation of vimentin expression; this leads to numerous phenotypic changes such as the loss of cellular adhesion and polarity and the acquisition of migratory and invasive phenotype [32]. In this study, we have found strong diffuse expression of vimentin by immunohistochemistry and concomitant loss of E-cad expression in the majority of poorly differentiated NSCLC but not in normal lung tissue and well-differentiated NSCLC. We have found that the level of co-expression of E-cad/vimentin was associated with lymph node metastasis (LNM) and poor pathological TNM stages and poor clinical survival. This could be explained by the frequency invasive subpopulations of tumor cells resulting from EMT. Decreased E-cad and increased vimentin expression in LNM correlated with a shorter DSS. Many studies have shown that EMT phenomenon indicated a short survival in many human tumors [16, 17, 33–39]. But some authors did not believe that vimentin was associated with survival in NSCLC [16].

We observed a correlation of KAI1 and E-cad protein downregulation with vessel infiltration and metastasis in NSCLC. In contrast, vimentin protein is upregulated in NSCLC. These indicated that KAI1 and EMT should be involved in tumor cell invasion and metastasis. On the whole sections, we found that the strong KAI1 and E-cadherin expression of tumor cells was far from stromal cells. Upregulation expression of vimentin in tumor cells was often close to and inside intratumoral vessels. At the same time, the level of KAI1 and E-cad expression, less than normal lung tissues, was associated with low or absent vimentin expression in the majority of well or moderately differentiated tumors. But in poorly differentiated tumors, weak and absent E-cad expression was associated with the predominated expression of cytoplasmic vimentin. This study also found that high expression of KAI1 and E-cad could inhibit tumor angiogenesis and lymphangiogenesis. Increased vimentin expression could promote tumor angiogenesis and lymphangiogenesis. KAI1 inhibited β-catenin tyrosine phosphorylation, and this would stabilize the E-cad functions [20]. These findings might indicate that KAI1 functions as a metastasis suppressor in the process of tumor invasion, angiogenesis, and lymphangiogenesis through inhibition of β-catenin-mediated EMT.

## Conclusions

It is suggested that KAI1 may play an important role in the LNM of NSCLC. Our results also show that KAI1 and EMT may be possible as a therapeutic marker for anti-metastasis therapy.

## Abbreviations

NSCLC: non-small cell lung cancer
EMT: epithelial-mesenchymal transition
MVD: microvessel density
LNM: lymph node metastasis
VI: vascular invasion
DSS: disease-specific survival
LVD: lymphatic vessel density
OS: overall
pTNM: pathological tumor-node-metastasis
IHC: immunohistochemistry
E-cad: E-cadherin
